# Characterization of a Hyaluronic Acid Utilization Locus and Identification of Two Hyaluronate Lyases in a Marine Bacterium *Vibrio alginolyticus* LWW-9

**DOI:** 10.3389/fmicb.2021.696096

**Published:** 2021-06-10

**Authors:** Xiaoyi Wang, Ziwei Wei, Hao Wu, Yujiao Li, Feng Han, Wengong Yu

**Affiliations:** ^1^Shandong Provincial Key Laboratory of Glycoscience and Glycoengineering, School of Medicine and Pharmacy, Ocean University of China, Qingdao, China; ^2^Laboratory for Marine Drugs and Bioproducts, Qingdao National Laboratory for Marine Science and Technology, Qingdao, China

**Keywords:** hyaluronate lyase, hyaluronic acid, polysaccharide utilization loci, *Vibrio*, *Proteobacteria*

## Abstract

Hyaluronic acid (HA) is a negatively charged and linear polysaccharide existing in the tissues and body fluids of all vertebrates. Some pathogenic bacteria target hyaluronic acid for adhesion and/or infection to host cells. *Vibrio alginolyticus* is an opportunistic pathogen related to infections of humans and marine animals, and the hyaluronic acid-degrading potential of *Vibrio* spp. has been well-demonstrated. However, little is known about how *Vibrio* spp. utilize hyaluronic acid. In this study, a marine bacterium *V. alginolyticus* LWW-9 capable of degrading hyaluronic acid has been isolated. Genetic and bioinformatic analysis showed that *V. alginolyticus* LWW-9 harbors a gene cluster involved in the degradation, transport, and metabolism of hyaluronic acid. Two novel PL8 family hyaluronate lyases, VaHly8A and VaHly8B, are the key enzymes for the degradation of hyaluronic acid. VaHly8A and VaHly8B have distinct biochemical properties, reflecting the adaptation of the strain to the changing parameters of the aquatic habitats and hosts. Based on genomic and functional analysis, we propose a model for the complete degradation of hyaluronic acid by *V. alginolyticus* LWW-9. Overall, our study expands our knowledge of the HA utilization paradigm within the *Proteobacteria*, and the two novel hyaluronate lyases are excellent candidates for industrial applications.

## Introduction

Animal cells are in close interaction with extracellular matrices (ECM), which function as a physical scaffold for organs and tissues, regulate various cellular functions and maintain homeostasis (Theocharis et al., 2016). Hyaluronic acid (HA), a significant constituent of ECM, is a linear polysaccharide consisting of repeating units of glucuronic acid and N-acetylglucosamine *via* a β-1,4 linkage. HA is involved in various physiological and pathological processes of the biological system, such as cell migration, adhesion, growth and differentiation, embryogenesis, cancer, inflammation, and damage repair (Volpi et al., 2009). Due to its excellent physicochemical characteristics, HA has a variety of applications in the pharmaceutical industry, such as orthopedics, ophthalmology, and aesthetic dermatology (Sudha and Rose, 2014).

Some pathogenic bacteria, such as *streptococci* and *streptobacillus*, produce extracellular or cell-surface hyaluronate lyase to depolymerize HA, facilitating the invasion of the host (Li and Jedrzejas, 2001; Oiki et al., 2017). Hyaluronate lyases degrade HA by β-elimination mechanism, generating unsaturated disaccharides with a C_4_-C_5_ double bond at the non-reducing end (Wang et al., 2017). Hyaluronate lyases are categorized into four polysaccharide lyase (PL) families, PL8, PL16, PL30, and PL33, in the Carbohydrate-Active Enzymes (CAZy) database according to primary structures (Lombard et al., 2014).

The utilization of HA requires multiple proteins, such as PLs, glycoside hydrolases (GHs), sugar transporters, and transcriptional factors. These genes often cluster in a polysaccharide utilization loci (PUL), orchestrating sensing, enzymatic digestion, transport, and metabolism of a specific polysaccharide (Martens et al., 2009; Grondin et al., 2017). There are some reports on the polysaccharide utilization locus of hyaluronic acid (PUL_HA_) in *Firmicutes* and *Fusobacteria*, but few reports on the PUL_HA_ in *Proteobacteria* (Kawai et al., 2018; Oiki et al., 2019a,b). Although several hyaluronate lyases of *Proteobacteria* have been characterized in detail, the pathway for HA utilization in *Proteobacteria* remains largely opaque (Han et al., 2014; Peng et al., 2018).

Members of the genus *Vibrio* are pathogenic bacteria that cause serious infections to aquatic animals and humans, called vibriosis (Austin, 2010). Vibriosis is one of the most common bacterial diseases posing a threat to cultured fish, shellfish, and shrimp, which has a negative effect on the development of the global aquaculture industry (Ina-Salwany et al., 2019). *Vibrio* infections occur when humans expose to contaminated water or consume raw or undercooked contaminated seafood, causing many diseases, such as gastroenteritis, and wound infections and septicemia (Dechet et al., 2008). *Vibrio* strains could degrade hyaluronic acid to facilitate host invasion; however, little is known about how they utilize hyaluronic acid.

In this study, we isolated a hyaluronate lyase-producing bacterium, *Vibrio alginolyticus* strain LWW-9. A PUL_HA_ was found in the draft genome of *V. alginolyticus* LWW-9 by genome analysis. In particular, two novel hyaluronate lyases in PUL_HA_, VaHly8A, and VaHly8B, were characterized. VaHly8A and VaHly8B showed distinct biochemical properties, which revealed their adaption to the living environment. Finally, we provided a model for how *V. alginolyticus* strain LWW-9 utilizes the HA. These results presented here not merely extend our understanding of the HA utilization paradigm within the *Proteobacteria* but also may contribute to the elucidation of bacterial physiology and pathogenicity.

## Materials and Methods

### Materials

Hyaluronic acid was obtained from Macklin (Shanghai, China). The pET-28a (+) plasmid and *Escherichia coli* BL21(DE3) were obtained from Takara (Dalian, China). DNA polymerase was obtained from Vazyme (Nanjing, China). Restriction enzymes and T_4_ DNA ligase were purchased from Takara (Dalian, China). Pageruler unstained protein ladder was obtained from Thermo Scientific (Wilmington, United States). All other chemicals were purchased from Sinopharm (Beijing, China).

### Isolation of Marine Hyaluronate Lyase-Producing Bacteria

Seawater was collected from Zhanqiao, Qingdao, China. A selective medium supplemented with HA as the sole carbon source was used to isolate hyaluronate lyase-producing bacteria from seawater. The medium consisted of 0.3% (w/v) KH_2_PO_4_, 0.7% (w/v) K_2_HPO_4_·3H_2_O, 0.2% (w/v; NH_4_)_2_SO_4_, 0.01% (w/v) MgSO_4_, 0.01% (w/v) FeSO_4_·7H_2_O, 3% NaCl, 0.05% (w/v) HA, and 1.5% (w/v) agar (pH 7.0). After microorganisms had grown at 25°C for 48 h, the plates were soaked with Gram’s iodine for 1 min (Patil and Chaudhari, 2017). Clones with distinct clearance zones were detected as HA-degrading strains. They were picked up and purified on the fresh selective medium plates for three times. The pure cultured strains were incubated at 25°C and 160 r/min for 48 h in 100 ml marine broth 2216, and the hyaluronate lyase activity in the culture supernatant was determined. The strain LWW-9 that exhibited the highest hyaluronate lyase activity was obtained and used in the following experiment.

### Identification of the Strain LWW-9

The 16S rDNA of strain LWW-9 was amplified by PCR using the universal primers 27F (5'-AGAGTTTGATCCTGGCTCAG-3') and 1492R (5'-TACGGTTACCTTGTTACGACTT-3'). A colony of strain LWW-9 was used as the template. The PCR product was purified, and sequenced by Ruibiotech Co., Ltd. (Beijing, China). The sequence analysis was conducted using Blast program^1^ to search for sequences with high identity in GenBank database. The phylogenetic analysis was performed by MEGA X using the neighbor-joining method (Kumar et al., 2018).

### Prediction of PUL_HA_ in *Vibrio alginolyticus* Strain LWW-9

The genomic DNA of strain LWW-9 was prepared using Tianamp bacteria DNA kit (Tiangen, China). The draft genome of strain LWW-9 was sequenced using Roche 454 FLX Titanium technologies (Margulies et al., 2005). The genome annotation was performed online in the Rapid Annotation using Subsystem Technology (RAST) server.^2^ Cazymes were further identified using pfam (Finn et al., 2014) and dbCAN Hidden Markov model (Zhang et al., 2018). Homologs searches of predicted protein sequences were carried out using Blatsp against NCBI PDB and nr databases. The gene cluster involved in the utilization of HA was identified as a potential PUL_HA_. If genes adjacent to hyaluronate lyases encoded proteins dedicated to the utilization of HA, including Cazymes, sugar transporters, and transcription factors, the boundary of PUL_HA_ was extended. When five continuous genes were not annotated as HA utilization proteins, the last gene with related function was regarded as the putative boundary of PUL_HA_.

### Sequence Analysis of VaHly8A and VaHly8B

The online Blastp algorithm was used to perform similarity searches against NCBI PDB and nr databases. Protein modules and domains were analyzed using Conserved Domain (CD) Search.^3^ A neighbor-joining tree based on the protein sequence alignment was constructed using MEGA X (Kumar et al., 2018). Amino acid alignment with other enzymes of the PL8 family was carried out using ESPrit 3.0 (Robert and Gouet, 2014). The physical and chemical parameters of proteins, such as molecular weight (Mw) and isoelectric point (*pI*) were predicted by the ProtParam tool on the ExPASy server.^4^ The existence and pattern of signal peptides were identified using SignalP 5.0 server.^5^

### Heterologous Expression and Purification of VaHly8A and VaHly8B

The *vahly8B* and *vahly8B* were amplified by PCR using the genomic DNA of strain LWW-9 as the template. The primers for VaHly8A were 5'-GGAATTCCATATGAATAAATTTAATATTTCAA-3' and 5'-CCGCTCGAGTTCCTTAATGCGTTTAAC-3'. The primers for VaHly8B were 5'-GGAATTCCATATGAAACCTCTGAAACTCAC-3' and 5'-CCGCTCGAGCTCTTTTACCAAAGAGAAGG-3'. The PCR products were recovered from the agarose gel, digested with *Nde* I and *Xho* I, and ligated into the expression plasmid pET-28a(+). The recombinant plasmids, pET28a(+)-VaHly8A and pET28a(+)-VaHly8B, were transformed into *E. coli* BL21(DE3) cells, respectively.

*Escherichia coli* BL21(DE3) cells harboring pET28a(+)-VaHly8A and pET28a(+)-VaHly8B were incubated in Luria-Bertani (LB) medium at 37°C until the OD_600_ reached 0.4–0.6, then induced with 0.02 mM isopropyl β-D-thiogalactoside at 18°C for 24 h. The cells were harvested by centrifugation, resuspended in 20 mM Na_2_HPO_4_-NaH_2_PO_4_ buffer (pH 7.4) containing 500 mM NaCl, and disrupted by sonication. The cell lysate was centrifuged, and the recombinant hyaluronate lyase with N-terminal and C-terminal (His)_6_ tags was purified from the supernatant by Histrap column (GE Healthcare, United States). The purity and Mw of the proteins were determined by sodium dodecyl sulfate-polyacrylamide gel electrophoresis (SDS-PAGE) on a 10% (w/v) resolving gel. Protein concentration was measured by the BCA protein assay kit (NCM Biotech, China).

### Enzyme Activity Assay

The enzyme activity was measured in a 1 ml reaction system under the optimal reaction condition. First, 0.1 ml enzyme (0.18 U/ml) was added to 0.9 ml 0.2% (w/v) HA substrate solution. After incubation for 10 min at the optimal temperature, the reaction was terminated by boiling for 10 min, and then the absorbance of the solution was measured at 232 nm by a UH5300 UV visible spectrophotometer (HITACHI, Japan). One unit of enzyme activity was defined as the amount of the protein required to produce 1 μmol unsaturated oligosaccharides using the molecular extinction coefficient value of 5,500 M^−1^ cm^−1^ at 232 nm (Lin et al., 1994).

### Biochemical Characterization of VaHly8A and VaHly8B

The optimal temperature was determined in 50 mM Tris-HCl buffer (pH 7.05) at different temperatures ranging from 0 to 70°C. The optimal pH was measured in the following buffers with various pH values: 50 mM Na_2_HPO_4_-Citrate buffer (pH 3.0–8.0), 50 mM NaH_2_PO_4_-Na_2_HPO_4_ buffer (pH 6.0–8.0), 50 mM Tris-HCl buffer (pH 7.05–8.95), and 50 mM Glycine-NaOH buffer (pH 8.6–10.6). To determine the thermostability of the enzyme, it was incubated for 1 h under temperatures ranging from 0 to 50°C, and the residual activities were determined at the optimal temperature and pH. To determine the pH stability of the enzyme, it was incubated for 6 h in buffers with varying pH values from 3.0 to 10.6 at 0°C, and the residual activities were determined at the optimal temperature and pH. The effect of NaCl was investigated by examining the enzyme activities in Tris-HCl buffer (pH 7.05) containing various concentrations of NaCl ranging from 0 to 1.0 M at the optimal temperature and pH. The effects of metal ions and surfactants were investigated by examining the enzyme activities in Tris-HCl buffer (pH 7.05) containing various compounds (1 mM) at the optimal temperature and pH.

### Kinetic Parameters of VaHly8A and VaHly8B

To investigate the kinetic parameters of VaHly8A and VaHly8B, 0.1–8.0 mg/ml HA were used as the substrate. 0.1 ml enzyme (0.36 U/ml) was added to 0.9 ml substrate solution. After incubation at the optimal temperature for 3 min, the absorbance of the solution was measured at 232 nm. *K*_m_ and *V*_max_ values were determined using the Michaelis-Menten equation and the curve fitting program by non-linear regression analysis using Graphpad Prism 8.

### Analysis of Degradation Pattern and Final Product

To investigate the degradation pattern and final product of HA by VaHly8A and VaHly8B, 0.2% (w/v) HA was digested by the purified enzyme (0.15 U/ml) at 20°C for VaHly8A and 30°C for VaHly8B. The reaction mixture was incubated for different time intervals ranging from 0 to 12 h. Samples were inactivated at 100°C for 10 min and centrifuged at 12,000 r/min for 10 min. The supernatant was then analyzed on a Superdex™ Peptide 10/300 GL column (GE Health, United States) by monitoring the absorbance at 232 nm. The mobile phase and flow rate were 0.2 M ammonium bicarbonate and 0.2 ml/min, respectively.

The exact Mw of final product was detected by negative ion electrospray ionization-mass spectroscopy (ESI-MS, Thermo Fisher Scientific, United States) with the mass acquisition range of 100–2,000. The ESI-MS analysis was carried out under the following conditions: sheath gas flow rate, 10 arb; spray voltage, 2.5 kV; tube lens, 35 V; capillary voltage, 16 V; and capillary temperature, 275°C.

## Results

### Identification of Strain LWW-9

**Figure 1:**
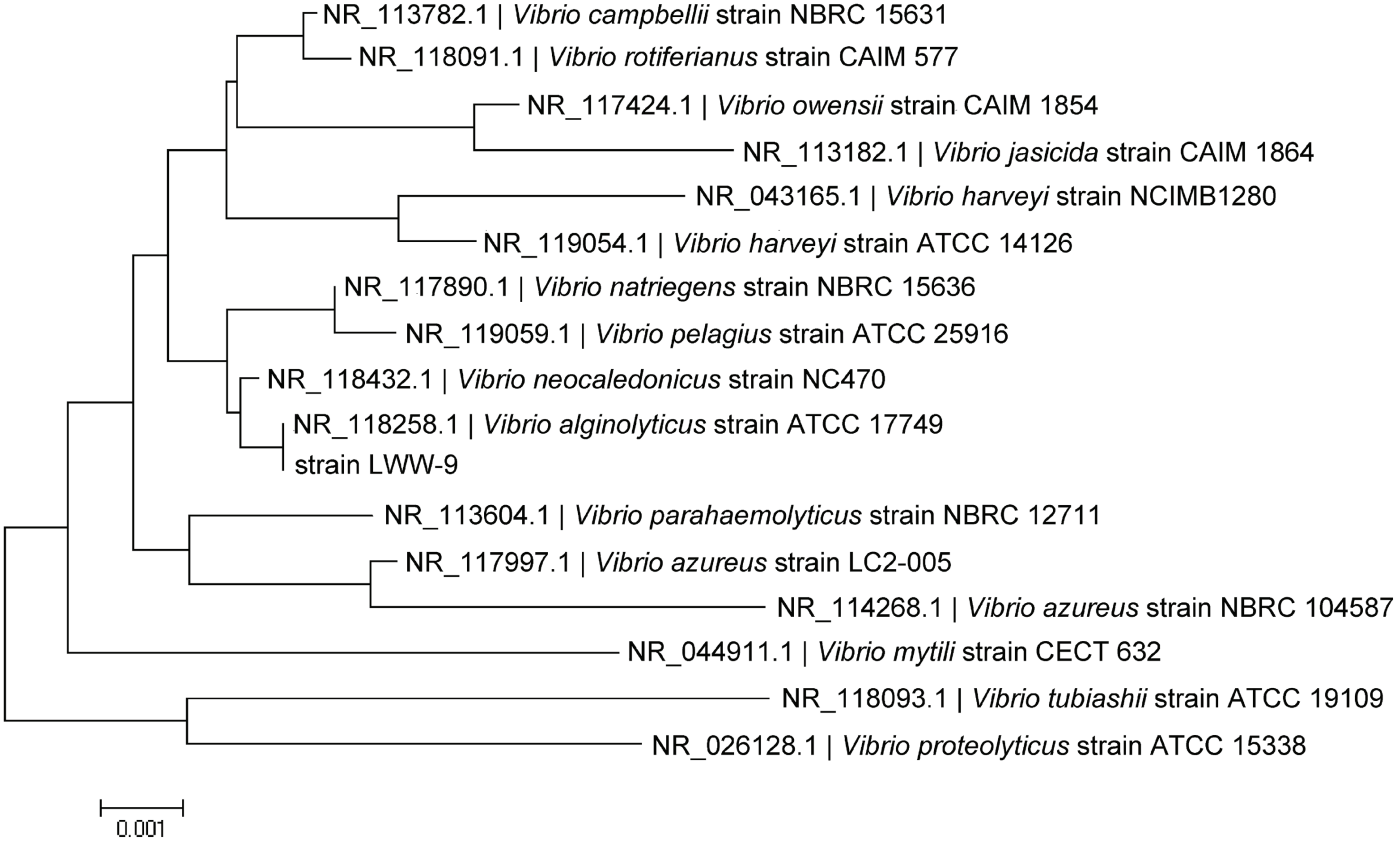
Phylogenetic tree of strain LWW-9 based on 16S rRNA sequences. The phylogenetic tree was generated by MEGA X using the neighbor-joining method.

The 16S rDNA of strain LWW-9 was sequenced and submitted to GenBank under the accession number MW396717. The Blast search analysis against GenBank database revealed that strain LWW-9 showed 99% identity with multiple *Vibrio* strains. *Vibrio alginolyticus* strain Va-X15 (MH298577.1) showed the highest identity of 99.23%. Sixteen type strains in *Vibrio* were selected for phylogenetic analysis, and the result showed that strain LWW-9 was closest to *V. alginolyticus* strain ATCC 17749 (NR_118258.1) in the phylogenetic tree (Figure 1). Therefore, strain LWW-9 was identified as *V. alginolyticus*.

### Model of HA Utilization in *V. alginolyticus* LWW-9

**Figure 2:**
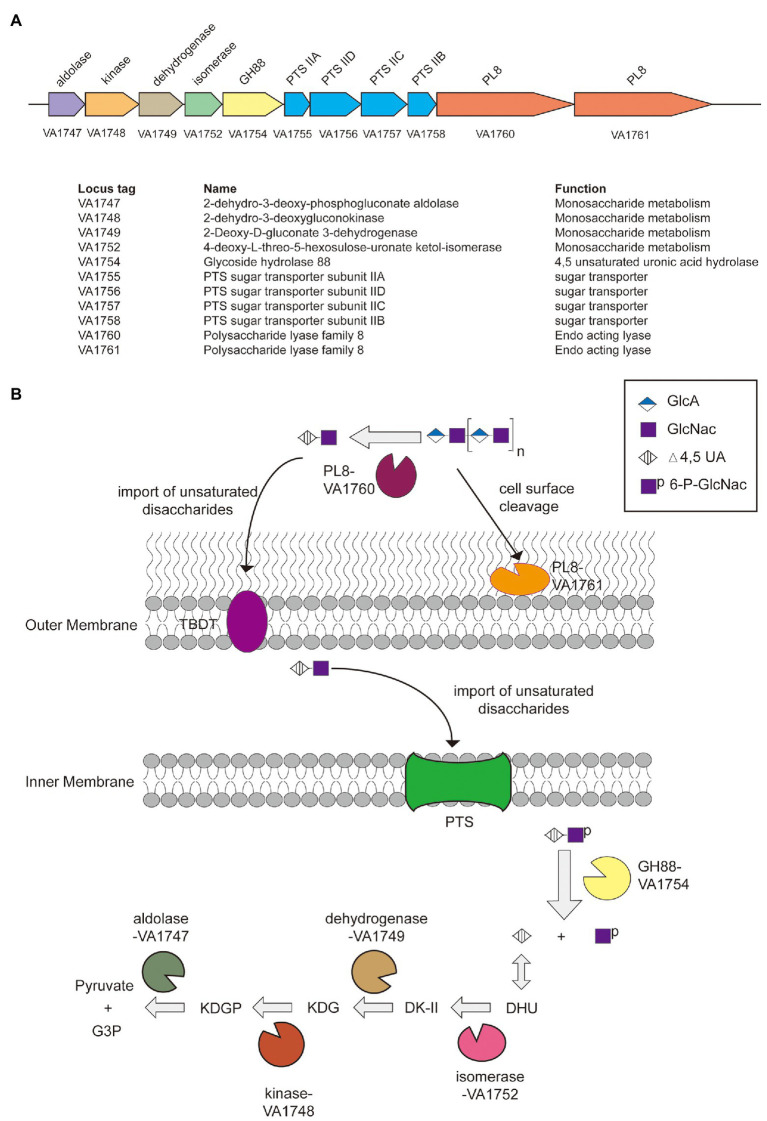
The paradigm of hyaluronic acid (HA) utilization by *Vibrio alginolyticus* LWW-9. **(A)** Predicted polysaccharide utilization locus of hyaluronic acid (PUL_HA_) in *V. alginolyticus* LWW-9. **(B)** Schematic of the cellular location, activity, and specificity of the PUL_HA_-encoded enzymes. DHU, 4-deoxy-L-*threo*-5-hexosulose-uronate; DK-II, 3-deoxy-D-*glycero*-2,5-hexodiulosonate; KDG, 2-keto-3-deoxy-D-gluconate; KDGP, 2-keto-3-deoxy-6-phosphogluconate; and G3P, glyceraldehyde-3-phosphate.

Genes related to the utilization of HA in *V. alginolyticus* LWW-9 were clustered in a ~19,600 bp genomic region, which suggested that this genetic cluster could be a PUL_HA_. The PUL_HA_ encodes two PL8 family hyaluronate lyases (VN1760 and VN1761), one GH88 family unsaturated glucuronyl hydrolase (VN1754), four enzymes involved in the metabolism of HA monosaccharides (VN1747, VN1748, VN1749, and VN1752), and one sugar transporter glucose phosphotransferase system (PTS) composed of four components (VN1755, VN1756, VN1757, and VN1758; Figure 2A). Despite the lack of *susC/susD* pairs in PUL_HA_, TonB-dependent transporter (TBDT) encoded elsewhere in the genome may enable the oligosaccharides sensing and transport, similar to the SusC/SusD system of *Bacteroides* (Blanvillain et al., 2007).

A pathway for the metabolism of HA in *V. alginolyticus* LWW-9 has been proposed (Figure 2B). HA is degraded to unsaturated disaccharides by extracellular and cell-surface hyaluronate lyases. Unsaturated disaccharides are first transported to the periplasm by TBDT and then imported to the cytoplasm by PTS. They are degraded to unsaturated uronates and N-acetyl-D-glucosamines by GH88 through hydrolysis of β-1,4 linkages in the cytoplasm. Unsaturated uronates are converted to 4-deoxy-L-*threo*-5-hexosulose-uronate (DHU) by nonenzymatic reactions. DHU was ultimately metabolized to pyruvate and glyceraldehyde-3-phosphate by consecutive reactions of isomerase, dehydrogenase, kinase, and aldolase (Maruyama et al., 2015).

### Sequence Analysis of VaHly8A and VaHly8B

The putative gene *vahly8A* was 2,385 bp in length and encoded VaHly8A consisting of 794 amino acid residues. The theoretical Mw and *pI* of VaHly8A are 88.1 kDa and 5.40, respectively. According to SignalP 5.0, VaHly8A has a type I signal peptide of 26 amino acid residues at its N-terminus. CD Search indicated that VaHly8A contained a Lyase_8 module (Trp^49^-Ile^381^) and a GAG lyase superfamily module (Phe^42^-Pro^740^). Blastp searches showed that VaHly8A shared the identity with HCLase (39%) from *Vibrio* sp. FC509 (Han et al., 2014), HAase-B (31%) from *Bacillus* sp. A50 (Guo et al., 2014), and XalA (30%) from *Paenibacillus alginolyticus* XL-1 (Ruijssenaars et al., 1999).

The putative gene *vahly8B* was 2,373 bp in length and encoded VaHly8B composed of 790 amino acid residues. The theoretical Mw and *pI* of VaHly8B are 86.5 kDa and 4.90, respectively. According to SignalP 5.0, VaHly8B has a type II signal peptide of 19 amino acid residues at its N-terminus. CD Search indicated that VaHly8A contained a Lyase_8 module (Trp^54^-Lys^373^) and a GAG_lyase superfamily module (Arg^51^-Ser^736^). Balstp searches showed that VaHly8B shared the identity with HCLase (41%) from *Vibrio* sp. FC509 (Han et al., 2014), XalA (33%) from *Paenibacillus alginolyticus* XL-1 (Ruijssenaars et al., 1999), and HAase-B (32%) from *Bacillus* sp. A50 (Guo et al., 2014).

**Figure 3:**
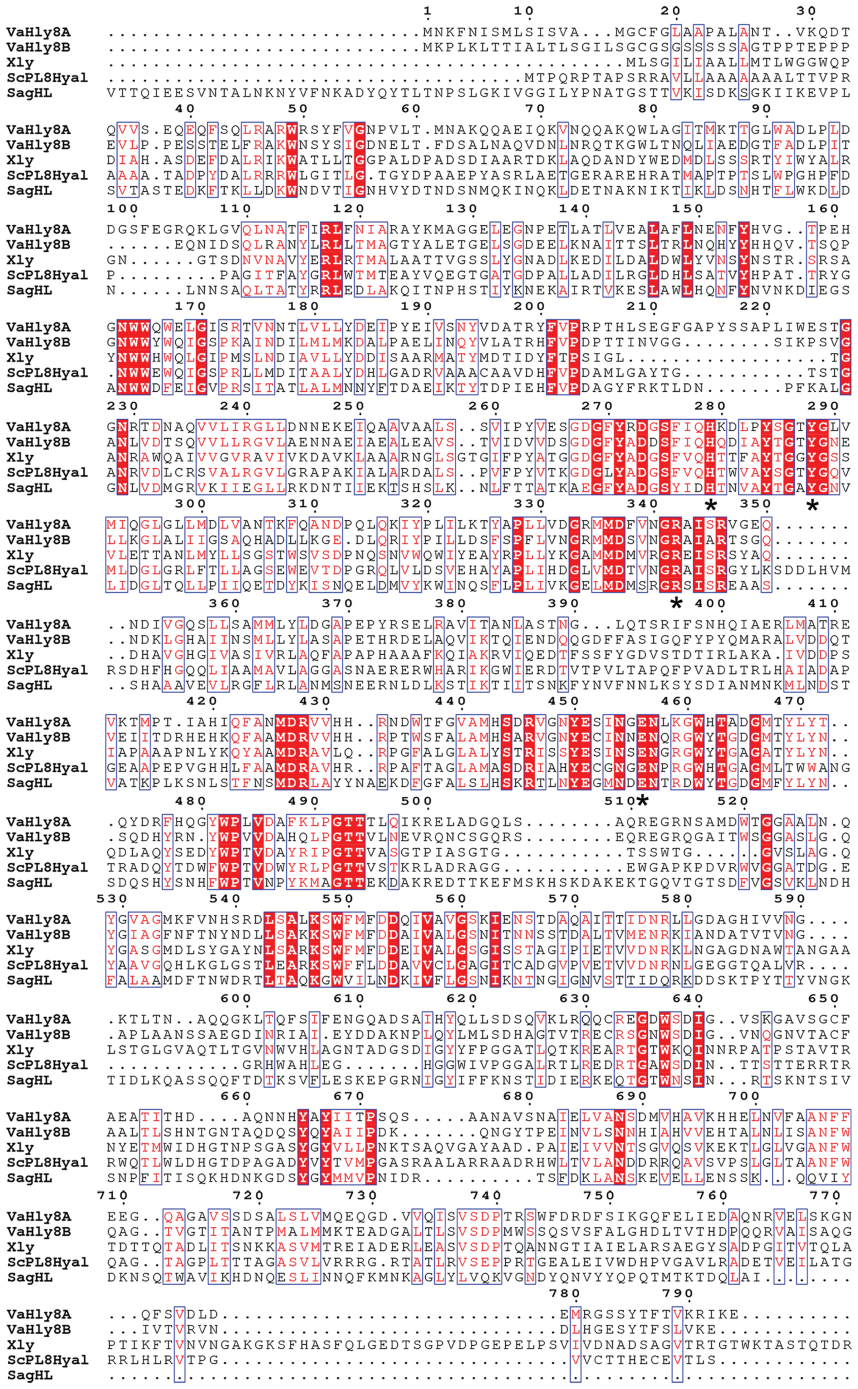
Protein sequence alignment of VaHly8A and VaHly8B to the identified enzymes of the PL8 family. Red background represents the same amino acid residues and blue frames indicate amino acid residues with identity > 70%. The critical catalytic residues were highlighted by asterisks below them. Xly, from *Bacillus* sp. GL1, Genbank: BAB21059.1; ScPL8Hyl, from *Streptomyces coelicolor* A3(2), Genbank: CAA19982.1; SagHL, from *Streptococcus agalactiae* NEM316, Genbank: CAD46929.1.

**Figure 4:**
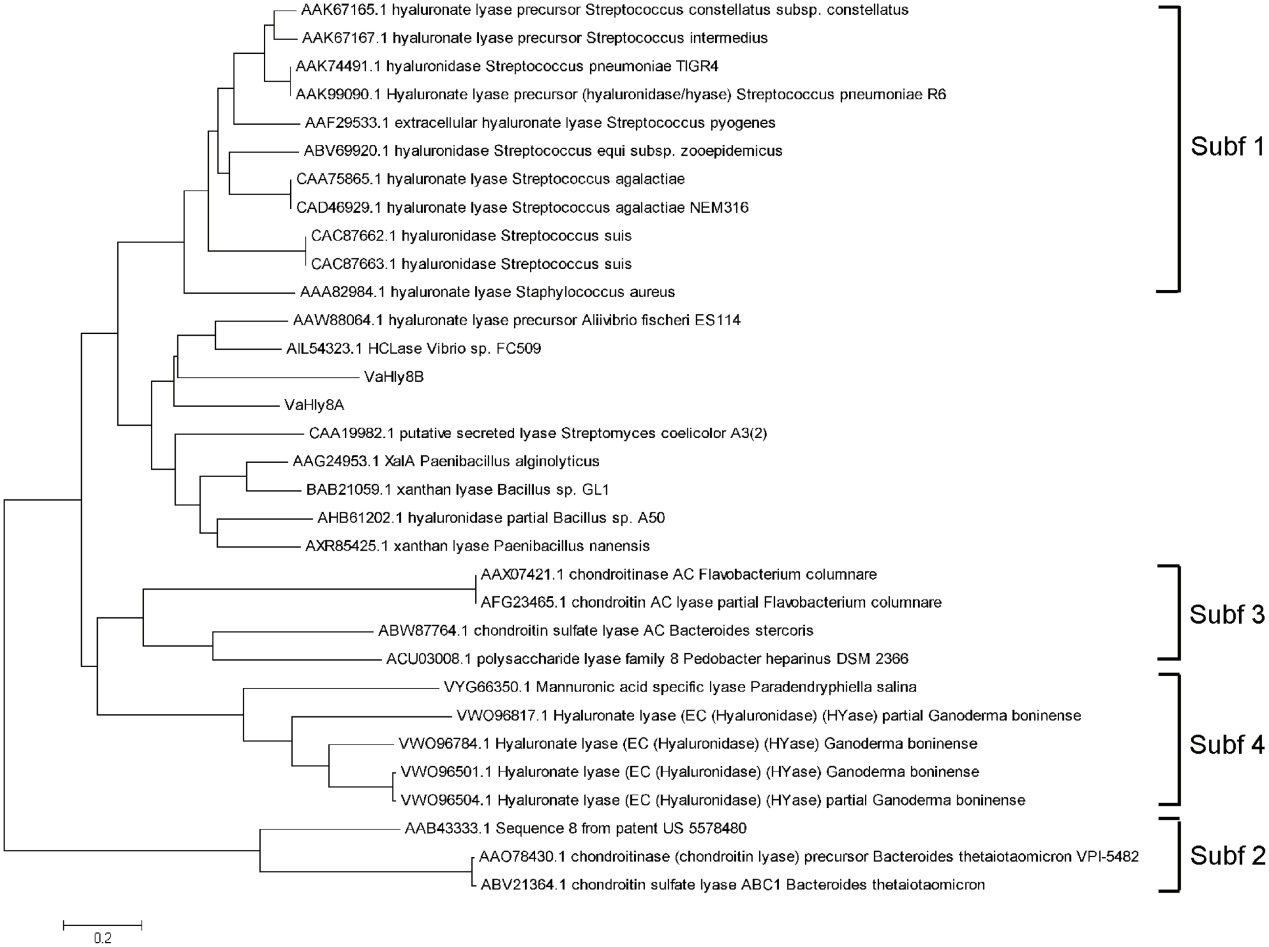
Phylogenetic tree of VaHly8A, VaHly8B and other enzymes of the PL8 family. The phylogenetic tree was constructed by MEGA X using the neighbor-joining method.

The amino acid alignment of VaHly8A, VaHly8B, and identified PL8 family enzymes showed that VaHly8A and VaHly8B contained the conserved catalytic residues of PL8 family (His^279^, Tyr^288^, Arg^342^, and Glu^456^ in VaHly8A; His^271^, Tyr^280^, Arg^333^, and Glu^451^ in VaHly8B; Figure 3). Phylogenetic tree was constructed and the result (Figure 4) revealed that VaHly8A and VaHly8B were new members of the PL8 family.

### Heterologous Expression of VaHly8A and VaHly8B in *E. coli*

**Figure 5:**
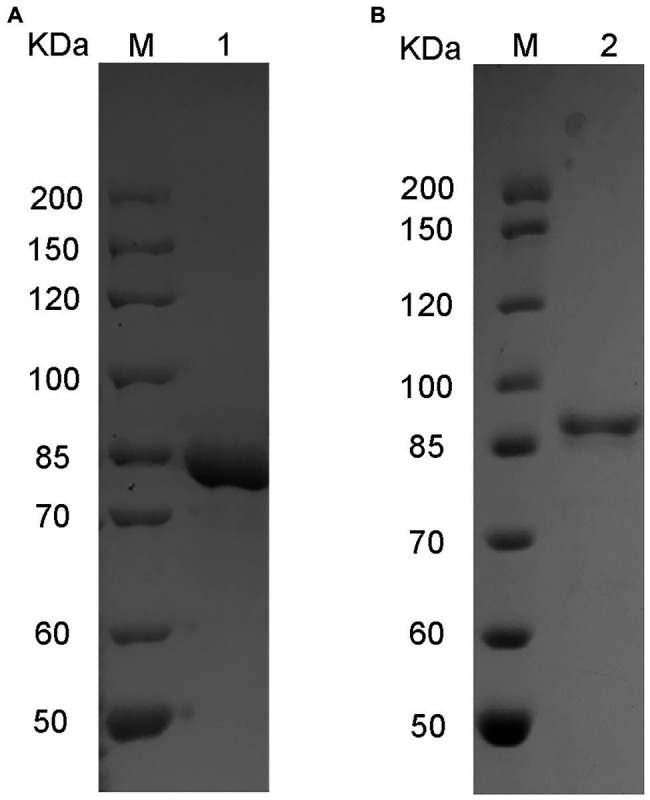
Sodium dodecyl sulfate-polyacrylamide gel electrophoresis (SDS-PAGE) of purified recombinant VaHly8A **(A)** and VaHly8B **(B)**. Lane M, unstained protein molecular weight marker; lane 1, purified VaHly8A; and lane 2, purified VaHly8B.

The genes *vahly8A* and *vahly8B* were heterologously expressed in pET-28a (+)/*E. coli* BL21(DE3) system and successfully purified by Ni-affinity chromatography. SDS-PAGE showed that VaHly8A (Figure 5A) and VaHly8B (Figure 5B) purified to homogeneity with Mw of approximately 83 and 87 kDa, respectively, which had no significant difference with the predicted Mw. The specific activity of VaHly8A and VaHly8B were 223.65 and 26.38 U/mg, respectively.

### Biochemical Properties of VaHly8A and VaHly8B

**Figure 6:**
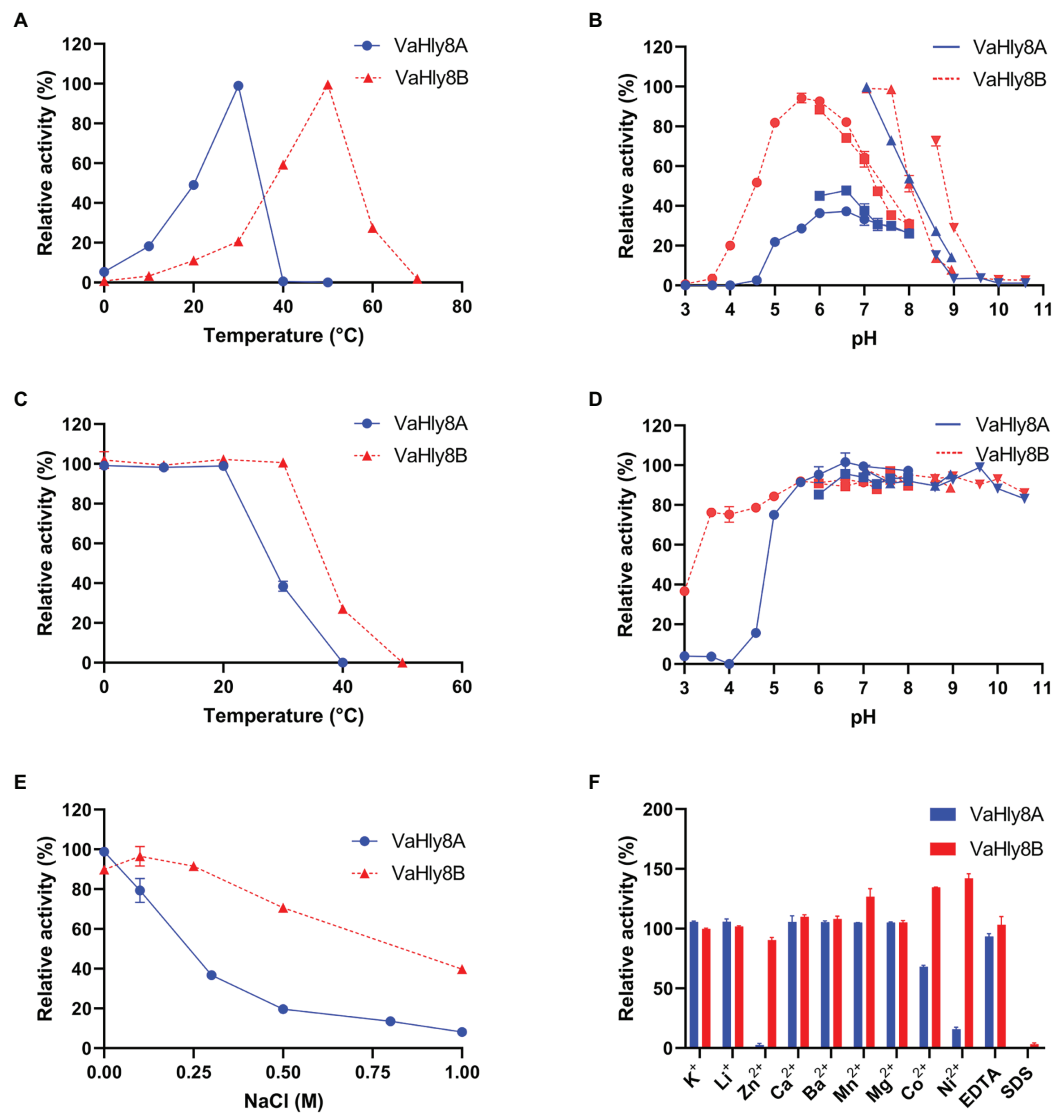
Biochemical properties of the hyaluronate lyases VaHly8A and VaHly8B. **(A)** Effect of temperature. The enzyme activities of VaHly8A (1.69 μg/ml) and VaHly8B (9.76 μg/ml) were measured at 0–70°C. The highest specific activity of VaHly8A (106.70 U/mg) at 30°C and VaHly8B (17.57 U/mg) at 50°C were set as 100%. **(B)** Effect of pH. The enzyme activities of VaHly8A (1.13 μg/ml) and VaHly8B (6.62 μg/ml) were measured in 50 mM buffers, including Na_2_HPO_4_-Citrate buffer (pH 3.0–8.0; filled circles), NaH_2_PO_4_-Na_2_HPO_4_ buffer (pH 6.0–8.0; filled squares), Tris-HCl buffer (pH 7.05–8.95; positive triangles), and Glycine-NaOH buffer (pH 8.6–10.6; inverted triangles). The highest specific activity of VaHly8A (223.65 U/mg) and VaHly8B (23.70 U/mg) in Tris-HCl buffer (pH 7.05) was set as 100%. **(C)** Thermostability of VaHly8A and VaHly8B. The enzymes were incubated for 1 h at different temperatures (0–50°C), and the residual activities were measured at 30°C for VaHly8A (5.83 μg/ml) and 50°C for VaHly8B (55.50 μg/ml). The initial specific activity of VaHly8A (223.65 U/mg) and VaHly8B (23.70 U/mg) were set as 100%. **(D)** The pH stability of VaHly8A and VaHly8B. The enzymes were incubated in above buffers (pH 3.0–10.60) for 6 h at 0°C, and the residual activities were measured at 30°C for VaHly8A (10.73 μg/ml) and 50°C for VaHly8B (61.75 μg/ml). The initial specific activity of VaHly8A (223.65 U/mg) and VaHly8B (23.70 U/mg) were set as 100%. **(E)** Effect of NaCl. The enzyme activities were measured in Tris-HCl buffer (pH 7.05) containing different concentrations of NaCl ranging from 0 to 1.0 M at 30°C for VaHly8A (0.67 μg/ml) and 50°C for VaHly8B (4.39 μg/ml). The highest specific activity of VaHly8A (223.65 U/mg) without NaCl and VaHly8B (26.38 U/mg) in the presence of 0.1 M NaCl were set as 100%. **(F)** Effects of various compounds. The enzyme activities were measured in Tris-HCl buffer (pH 7.05) containing 1 mM various compounds at 30°C for VaHly8A (1.06 μg/ml) and 50°C for VaHly8B (3.60 μg/ml). The specific activity of VaHly8A (223.65 U/mg) and VaHly8B (26.38 U/mg) without tested compounds was set as 100%. Values represent the mean of three replicates ± SD.

VaHly8A exhibited the maximal activity at 30°C (Figure 6A) and maintained over 90% original activity after incubation at temperatures from 0 to 20°C for 1 h (Figure 6C). VaHly8B showed the highest activity at 50°C (Figure 6A) and retained over 90% original activity after incubation at temperatures from 0 to 30°C for 1 h (Figure 6C). Compared with VaHly8B, VaHly8A had a lower optimal temperature and thermostability. The optimal pH of VaHly8A and VaHly8B was 7.05 in Tris-HCl buffer (Figure 6B). VaHly8A retained over 70% original activity after incubation at pH ranging from 5.0 to 10.6 for 6 h (Figure 6D). VaHly8B maintained over 70% original activity after incubation at pH ranging from 3.6 to 10.6 for 6 h. Despite the same optimal pH of VaHly8A and VaHly8B, VaHly8B showed higher activity and stability than VaHly8A under acidic conditions. The activity of VaHly8A was inhibited in the presence of NaCl (Figure 6E). However, VaHly8B is more tolerant of NaCl than VaHly8A, and the activity of VaHly8B reached the maximum when the concentration of NaCl was 100 mM. Mn^2+^, Co^2+^, and Ni^2+^ showed significantly stimulating effects on VaHly8B with 126.8, 134.5, and 142.0% of relative activity, respectively (Figure 6F). The activity of VaHly8A was not obviously enhanced by these metal ions, but inhibited by Co^2+^ and Ni^2+^. The activities of both VaHly8A and VaHly8B were strongly inhibited by SDS. Besides, the activity of VaHly8A was strongly inhibited by Zn^2+^. Other tested chemicals had no significant effect on both VaHly8A and VaHly8B. Overall, VaHly8B had higher resistance to metal ions than VaHly8A. As shown in Table 1, the *K_m_* and *k_cat_* of VaHly8A toward HA were 1.21 μM and 477.93 s^−1^, respectively. The *K_m_* and *k_cat_* of VaHly8B toward HA were 0.78 μM and 54.59 s^−1^, respectively.

**Table 1:** Specific activity and kinetic parameters of VaHly8A and VaHly8B.

	Specific Activity (U·mg^−1^)	*V_max_* (μΜ·min^−1^)	*K_m_* (μM)	*K_cat_* (s^−1^)	*k_cat_/K_m_* (s^−1^·μM^−1^)
VaHly8A	223.65	10.61 ± 0.46	1.21 ± 0.14	477.93 ± 20.52	394.66 ± 62.61
VaHly8B	26.38	0.10 ± 0.00	0.78 ± 0.06	54.59 ± 1.18	69.98 ± 6.89

### Degradation Patterns and End Products of VaHly8A and VaHly8B

**Figure 7:**
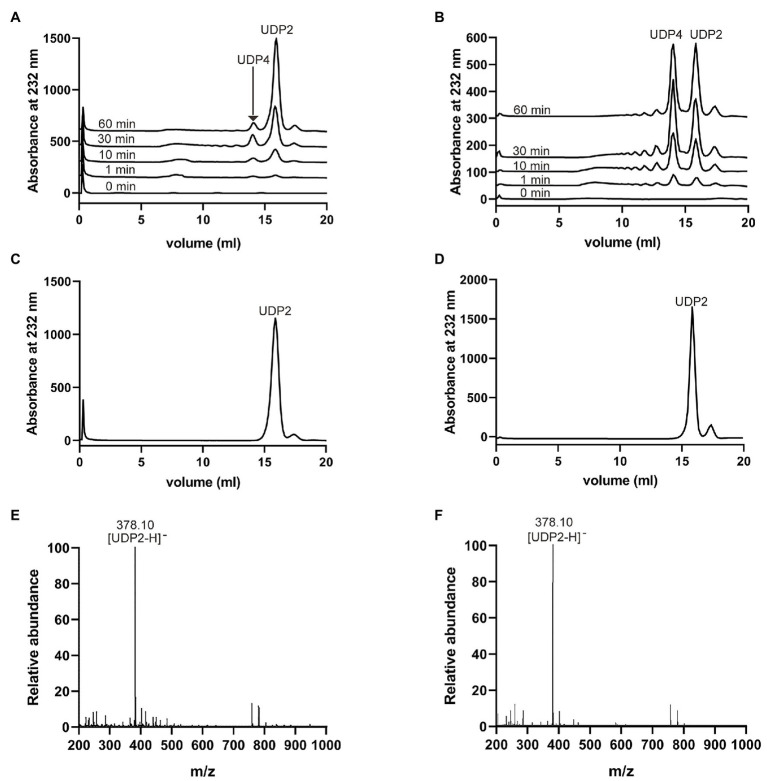
Action modes and final products of VaHly8A and VaHly8B. **(A)** Time-course treatment of HA using VaHly8A at 30°C. **(B)** Time-course treatment of HA using VaHly8B at 50°C. Analysis of the final products of HA digested by VaHly8A **(C)** and VaHly8B **(D)** using gel filtration chromatography with a Superdex™ peptide 10/300 gel filtration column. Electrospray ionization-mass spectroscopy (ESI-MS) analysis of the final products of HA digested by VaHly8A **(E)** and VaHly8B **(F)**.

To investigate the degradation patterns of VaHly8A and VaHly8B, reaction products incubated for different time intervals were analyzed by the Superdex™ peptide 10/300 gel filtration column. The appearance of unsaturated oligosaccharides was detected using the absorbance at 232 nm. At the beginning of the reaction, products with high degree of polymerization were produced (Figures 7A,B). As the reaction continues, smaller oligomers continuously accumulated. The HA was completely digested after 6 h by VaHly8A and 12 h by VaHly8B (Figures 7C,D). These results indicated that both VaHly8A and VaHly8B acted in an endolytic manner.

To further obtain the exact molecular weight of the final products, the negative-ion ESI-MS was used (Figures 7E,F). Both main peaks in mass spectra were 378.10 m/z, corresponding to the molecular weight of unsaturated disaccharides. Therefore, VaHly8A and VaHly8B degraded HA to unsaturated disaccharides as the final products.

## Discussion

Hyaluronic acid-degrading bacteria are common in the marine ecosystem: a few studies previously reported hyaluronate lyase-encoding *bacilli* (Kurata et al., 2015) and *gammaproteobacterial* (Han et al., 2014; Peng et al., 2018). In this study, a hyaluronate lyase-producing marine bacterium, *V. alginolyticus* LWW-9, was isolated from seawater.

Based on the bioinformatic analysis, we discovered an enzymatic HA degradation system in *V. alginolyticus*. The organization of PUL_HA_ of *V. alginolyticus* closely resembles the HA PULs in *Firmicutes* and *Fusobacteria* (Oiki et al., 2017, 2019b; Kawai et al., 2018). However, compared with archetypal PULs of *Bacteroides*, PUL_HA_ lacks *susC/susD* pairs encoding a TBDT and a glycan-binding lipoprotein, respectively (Tancula et al., 1992; Reeves et al., 1997). SusC/SusD-like proteins are considered as the hallmark of PUL and have been used to identify PULs in the genomes of *Bacteroides*. In the genome of *V. alginolyticus*, no protein showing similarity with SusD was detected. Similar to other bacteria in *Proteobacteria*, *V. alginolyticus* contains TBDT proteins in the genome, which is the counterpart of SusC/SusD pairs in *Proteobacteria* (Blanvillain et al., 2007; Neumann et al., 2015). Blastp searches revealed that none of TBDTs identified in *V. alginolyticus* genome displayed high similarity with SusC. These results strongly support Blanvillain’s opinion that TBDTs related to glycan uptake evolved independently in *Proteobacteria* and *Bacteroidetes* (Blanvillain et al., 2007).

The combination of genomic studies and biochemical characterizations of individual CAZymes can enhance our knowledge of the functions of PULs in microbial communities. Here, two hyaluronate lyases in the PUL_HA_, VaHly8A, and VaHly8B, were heterologously expressed, purified, and characterized. VaHly8A has a type I signal peptide, whereas VaHly8B has a type II signal peptide, suggesting their different subcellular localization in the bacterial cells. Hence, VaHly8A is an extracellular enzyme, whereas VaHly8B is an outer membrane enzyme. Moreover, they show distinct biochemical properties. The survival and colonization of vibrios depend on their adaption to variable parameters of the aquatic habitats and respective hosts. From the perspective of evolution, the generation of these two hyaluronate lyases is the result of the strain’s adaptation to environmental changes.

VaHly8A and VaHly8B exhibited the highest activity at 30 and 50°C, respectively. By contrast, the optimal temperatures of most identified enzymes of PL8 are 37–45°C (Table 2). VaHly8A was a cold-adapted hyaluronate lyase with lower optimal temperature and thermostability, which can conserve energy and reduce the risk of environmental contamination. Furthermore, it can be inactivated selectively by increasing the temperature slightly. Owing to these properties, the enzymatic reaction can be easily terminated and the product can be conveniently separated from the reaction mixture. Both VaHly8A and VaHly8B are most active at neutral pH, which is different from most characterized hyaluronate lyases from the PL8 family with the highest activity at acidic conditions (Table 2). VaHly8B retained about 70% activity at pH 3.6–10.6. Compared with most identified enzymes of PL8 (Table 2), VaHly8B was stable over a wider pH range. This property is advantageous for the storage of the enzyme preparation. Except SDS, most metal ions and EDTA did not obviously inhibit the activity of VaHly8B. The result revealed that VaHly8B was resistant to many metal ions. VaHly8B can degrade HA in the complex environment, which is beneficial to its industrial application. The specific activity of hyaluronate lyases is generally tens to hundreds of units per milligram by A_232_ enzyme activity assay, such as HCLase Er (13.8 U/mg) from *Vibrio* sp. FC509 (Peng et al., 2018), BniHL (136.7 U/mg) from *Bacillus niacin* (Kurata et al., 2015), and HAase (292.7 U/mg) from *Arthrobacter globiformis* A152 (Zhu et al., 2017a). In comparison, VaHly8A exhibited a higher specific activity. Our findings indicated that VaHly8A and VaHly8B are two hyaluronate lyases with novel enzymatic properties.

**Table 2:** Comparison of the biochemical properties of VaHly8A and VaHly8B with other PL8 family enzymes.

Enzyme	Source	Optimal temperature (°C)	Optimal pH	pH stability	References
VaHly8A	*Vibrio alginolyticus* LWW-9	30	7.05	5.6–10.6	This study
VaHly8B	*Vibrio alginolyticus* LWW-9	50	7.05	3.6–10.6	This study
HylB	*Streptococcus zooepidemicus* ATCC39920	37	6	N/A	Sun et al., 2015
HAase	*Arthrobacter globiformis* A152	42	4	4–10	Zhu et al., 2017a
HAase-B	*Bacillus* sp. A50	44	6.5	5–7	Guo et al., 2014
BniHL	*Bacillus niacini*	45	6	6–10	Kurata et al., 2015
ScPL8H	*Streptomyces coelicolor* A3(2)	57	5.2	N/A	Elmabrouk et al., 2011
HCLase	*Vibrio* sp. FC509	30	8	N/A	Han et al., 2014
Vpa_0049	*Vibrio* sp. QY108	30	8	7–10.6	Zhang et al., 2020
HCLaseM	*Microbacterium* sp. H14	40	7	5–9	Sun et al., 2019
ChSase ABC	*Acinetobacter* sp. C26	42	6	5–10	Zhu et al., 2017b
ChonABC	Bacteroides thetaiotaomicron WAL2926	37	7.6	N/A	Shaya et al., 2008
ChSase ABC	*Sphingomonas paucimobilis*	40	6.5	N/A	Fu et al., 2018
cABC I	*Proteus vulgaris*	37	8	N/A	Hamai et al., 1997
AsChnAC	*Arthrobacter* sp.	37	7.2	N/A	Yin et al., 2016
ChSase AC	*Flavobacterium heparinum*	40	6.8	N/A	Gu et al., 1995
ChSase AC II	*Arthrobacter* sp. CS01	37	6.5	4.5–8.5	Fang et al., 2019
ChSase AC	*Bacteroides stercoris*	45–50	5.7–6.0	N/A	Hong et al., 2002

N/A, There are no clear data in the reference.

Hyaluronic acid exists extensively in diverse connective tissues and the nervous system of virtually all animals. *Vibrio alginolyticus*, a common pathogenic marine *Vibrio* species, is not only an emerging pathogen inducing human infection but also a common cause of economic loss in the aquaculture industry (Cao et al., 2018). *Vibrio alginolyticus* secrets extracellular and cell-surface hyaluronate lyases to degrade HA, leading to the breakdown of biophysical barrier of the host tissues and exposure of host cells to bacterial toxins. The degradation of HA promotes the invasion and spreading of *V. alginolyticus* in the host. Therefore, the PUL_HA_ of *V. alginolyticus* reflects the bacterial ability to utilize the given glycan as a nutrient source for survival and to produce the “spreading factors” hyaluronate lyases for colonization.

Currently, antibiotics have been mainly used to resolve *V. alginolyticus*-related diseases (Grimes, 2020). However, the long-term use of antibiotics may result in harmful consequences, such as antibiotic residues and drug resistance (Langdon et al., 2016). Thus, finding an effective alternative method to regulate *V. alginolyticus* infection is highly significant. The functional characterization of PUL_HA_ broadens our knowledge about the physiology and pathogenicity of *V. alginolyticus* and enables the development of novel preventive and therapeutic strategies against *V. alginolyticus*-associated infection.

In summary, we reported the discovery and characterization of a PUL that orchestrates the utilization of HA in a marine bacterium *V. alginolyticus* LWW-9. The PLs, GH, and enzymes related to monosaccharide metabolism encoded by PUL_HA_ provide an example of how *V. alginolyticus* completely degrade HA. The presence of two novel hyaluronate lyases with distinct biochemical properties provides critical insights into how *V. alginolyticus* adapts to variable parameters of the aquatic habitats and hosts for survival and colonization. Our report strengthens the previous proposition (Blanvillain et al., 2007) that TBDTs related to glycan uptake evolved independently in *Proteobacteria* and *Bacteroidetes*. Furthermore, the functional characterization of PUL_HA_ facilitates the illustration of physiology and pathogenicity of *V. alginolyticus* and promotes the development of alternative non-antibiotic-based means of controlling bacterial infections.

## Data Availability Statement

The datasets presented in this study can be found in online repositories. The names of the repository/repositories and accession number(s) can be found at: NCBI (accession: MW396717).

## Author Contributions

WY and FH: conceptualization. XW: methodology, investigation, and writing – original draft preparation. ZW and YL: investigation and data curation. HW: software and data curation. All authors contributed to the article and approved the submitted version.

### Conflict of Interest

The authors declare that the research was conducted in the absence of any commercial or financial relationships that could be construed as a potential conflict of interest.

## Footnotes

^1^http://blast.ncbi.nlm.nih.gov/Blast.cgi

^2^https://rast.nmpdr.org/

^3^https://www.ncbi.nlm.nih.gov/Structure/cdd/wrpsb.cgi

^4^https://web.expasy.org/protparam/

^5^http://www.cbs.dtu.dk/services/SignalP/
