# Supplementary material for: Characterization of a Hyaluronic Acid Utilization Locus and Identification of Two Hyaluronate Lyases in a Marine Bacterium Vibrio alginolyticus LWW-9
Source: Front Microbiol. 2021 Jun 10;12:696096. doi: 10.3389/fmicb.2021.696096 (PMC8222515; doi:10.3389/fmicb.2021.696096)
Supplement: Supplementary file 1 [file Table_1.DOCX]

Supplementary Material


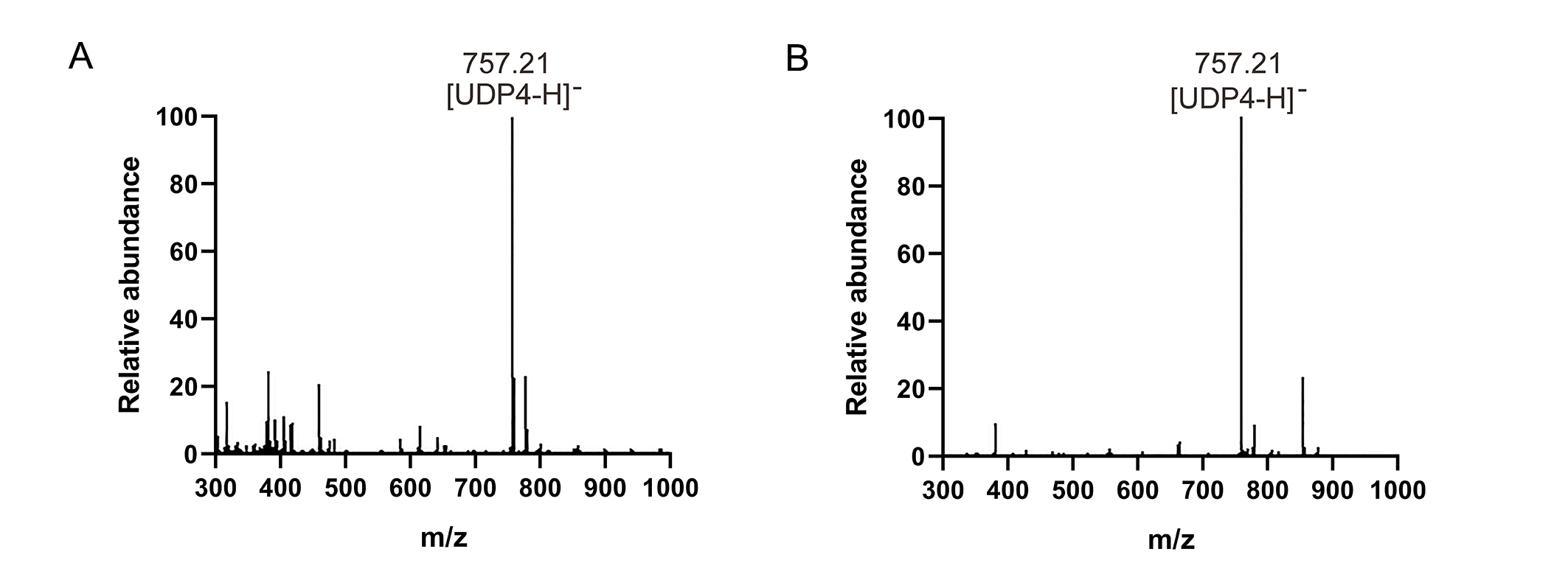


**Supplementary Figure 1.** ESI-MS analysis of the tetrasaccharides of HA digested by VaHly8A (A) and VaHly8B (B).


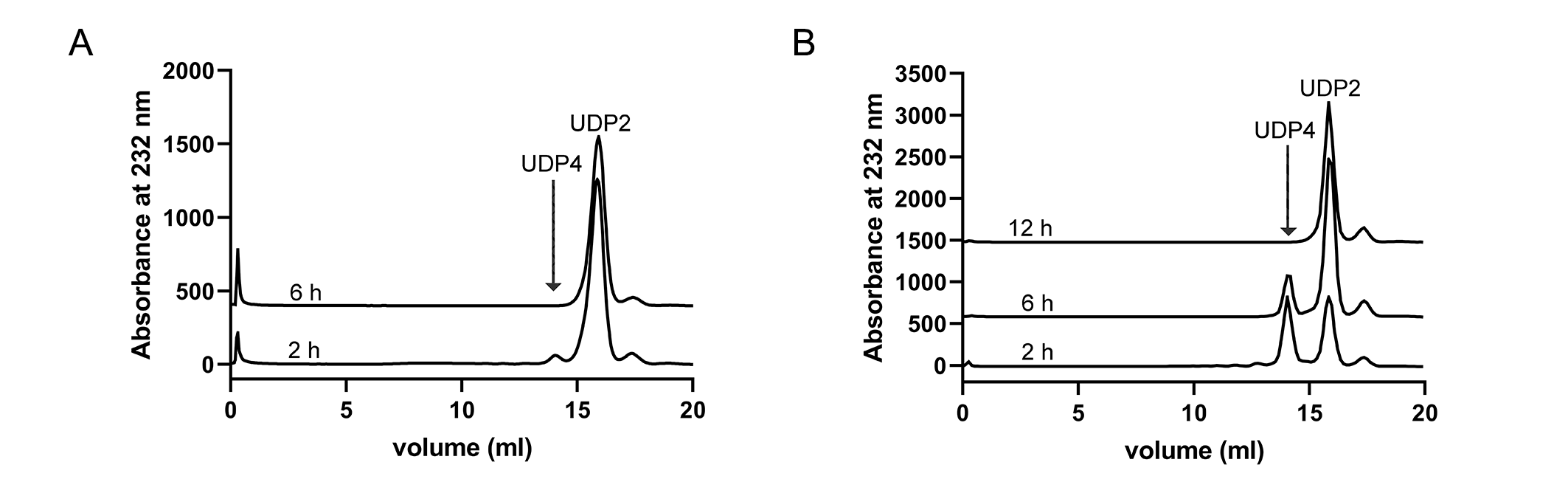


**Supplementary Figure 2.** (A) Time-course treatment of HA using VaHly8A at 2 h and 6 h. (B) Time-course treatment of HA using VaHly8B at 2 h, 6 h and 12 h.
